# Multiplex protein profiling of bronchial aspirates reveals disease-, mortality- and respiratory sequelae-associated signatures in critically ill patients with ARDS secondary to SARS-CoV-2 infection

**DOI:** 10.3389/fimmu.2022.942443

**Published:** 2022-07-29

**Authors:** Marta Molinero, Silvia Gómez, Iván D. Benítez, J. J. Vengoechea, Jessica González, Dinora Polanco, Clara Gort-Paniello, Anna Moncusí-Moix, María C. García-Hidalgo, Manel Perez-Pons, Thalía Belmonte, Gerard Torres, Jesús Caballero, Carme Barberà, Jose Ignacio Ayestarán Rota, Lorenzo Socías Crespí, Adrián Ceccato, Laia Fernández-Barat, Ricard Ferrer, Dario Garcia-Gasulla, Jose Ángel Lorente-Balanza, Rosario Menéndez, Ana Motos, Oscar Peñuelas, Jordi Riera, Antoni Torres, Ferran Barbé, David de Gonzalo-Calvo

**Affiliations:** ^1^ Translational Research in Respiratory Medicine, University Hospital Arnau de Vilanova and Santa Maria, IRBLleida, Lleida, Spain; ^2^ CIBER of Respiratory Diseases (CIBERES), Institute of Health Carlos III, Madrid, Spain; ^3^ Intensive Care Department, University Hospital Arnau de Vilanova, IRBLleida, Lleida, Spain; ^4^ Intensive Care Department, University Hospital Santa María, IRBLleida, Lleida, Spain; ^5^ Intensive Care Unit, Son Espases University Hospital, Instituto de Investigación Sanitaria Illes Balears (IdISBa), Palma de Mallorca, Spain; ^6^ Critical Care Department, Son Llàtzer Hospital, Palma de Mallorca, Spain; ^7^ Servei de Pneumologia, Hospital Clinic, Universitat de Barcelona, IDIBAPS, Barcelona, Spain; ^8^ Intensive Care Department, Vall d’Hebron Hospital Universitari. SODIR Research Group, Vall d’Hebron Institut de Recerca VHIR), Barcelona, Spain; ^9^ Barcelona Supercomputing Center (BSC), Barcelona, Spain; ^10^ Hospital Universitario de Getafe, Madrid, Spain; ^11^ Pulmonology Service, University and Polytechnic Hospital La Fe, Valencia, Spain

**Keywords:** acute respiratory distress syndrome, bronchial aspirate, COVID-19, proteomics, ICU – intensive care unit

## Abstract

**Introduction:**

Bronchial aspirates (BAS) obtained during invasive mechanical ventilation (IMV) constitutes a useful tool for molecular phenotyping and decision making.

**Aim:**

To identify the proteomic determinants associated with disease pathogenesis, all-cause mortality and respiratory sequelae in BAS samples from critically ill patients with SARS-CoV-2-induced ARDS

**Methods:**

Multicenter study including 74 critically ill patients with COVID-19 and non-COVID-19 ARDS. BAS were obtained by bronchoaspiration after IMV initiation. Three hundred sixty-four proteins were quantified using proximity extension assay (PEA) technology. Random forest models were used to assess predictor importance.

**Results:**

After adjusting for confounding factors, CST5, NADK, SRPK2 and TGF-α were differentially detected in COVID-19 and non-COVID-19 patients. In random forest models for COVID-19, CST5, DPP7, NADK, KYAT1 and TYMP showed the highest variable importance. In COVID-19 patients, reduced levels of ENTPD2 and PTN were observed in nonsurvivors of ICU stay, even after adjustment. AGR2, NQO2, IL-1α, OSM and TRAIL showed the strongest associations with in-ICU mortality and were used to construct a protein-based prediction model. Kaplan-Meier curves revealed a clear separation in mortality risk between subgroups of PTN, ENTPD2 and the prediction model. Cox regression models supported these findings. In survivors, the levels of FCRL1, NTF4 and THOP1 in BAS samples obtained during the ICU stay correlated with lung function (i.e., D_LCO_ levels) 3 months after hospital discharge. Similarly, Flt3L and THOP1 levels were correlated with radiological features (i.e., TSS). These proteins are expressed in immune and nonimmune lung cells. Poor host response to viral infectivity and an inappropriate reparative mechanism seem to be linked with the pathogenesis of the disease and fatal outcomes, respectively.

**Conclusion:**

BAS proteomics identified novel factors associated with the pathology of SARS-CoV-2-induced ARDS and its adverse outcomes. BAS-based protein testing emerges as a novel tool for risk assessment in the ICU.

## Introduction

Acute respiratory distress syndrome (ARDS) secondary to SARS-CoV-2 infection is a hallmark of severe clinical courses of COVID-19 (1–4). Patients admitted to the intensive care unit (ICU) show mortality rates exceeding 25-30% (5), particularly among those requiring invasive mechanical ventilation (IMV) (6). Despite the effectiveness of COVID-19 vaccines (7, 8), ICU admission of patients with SARS-CoV-2-induced ARDS remains a challenge for health systems (9). The appearance of novel contagious SARS-CoV-2 strains should not be discarded (10). Novel insights into the mechanisms involved in the disease and its outcomes are important for designing therapeutic strategies to improve ICU management. The prediction of the patient’s evolution and postacute sequelae would anticipate the course of the disease. In this scenario, one potential approach is to analyze a large number of biomarkers that participate in disease pathways and/or reflect pathophysiological mechanisms, e.g., proteomic tools.

During IMV, bronchoscopies are routinely performed as part of standard care. This procedure allows for the collection of bronchial aspirates (BAS), which can be informative about the local environment of the lower respiratory tract. Compared to other respiratory specimens, such as bronchoalveolar lavage fluid (BALF), BAS collection causes fewer hemodynamic and respiratory complications. In addition to its utility for microbiological sampling, BAS shows a high diagnostic yield and constitutes a useful tool in therapeutic decision making (11, 12). Altogether, BAS emerges as a valuable matrix for molecularly phenotyping those patients with ARDS (13).

Although previous studies have demonstrated that viral infections, including SARS-CoV-2, impact the molecular fingerprint of respiratory samples (14, 15), the analysis of BAS to evaluate the host response to pathogens has been poorly addressed. Above and beyond the biomarker value, the proteomic bioprofile could reflect pathophysiological processes linked to SARS-CoV-2 infection and its progression to adverse outcomes (16). The current investigation analyzed the proteomic signature of BAS samples from patients with ARDS secondary to SARS-CoV-2. First, we sought to characterize the protein signature induced by SARS-CoV-2 infection in patients under IMV. Second, we explored the proteomic determinants linked to in-ICU mortality and lung sequelae in patients with ARDS secondary to SARS-CoV-2 infection. The final aim was to define the host response to the infection and to identify biological pathways and proteomic biomarkers associated with adverse outcomes and postacute sequelae in the critical clinical courses of the disease. To our knowledge, this is the first study that implements extensive targeted protein profiling in BAS specimens obtained from COVID-19 or non-COVID-19 ARDS patients under IMV and links the bioprofiles to their follow-ups.

## Methods

### Study design and patients

**Figure 1:**
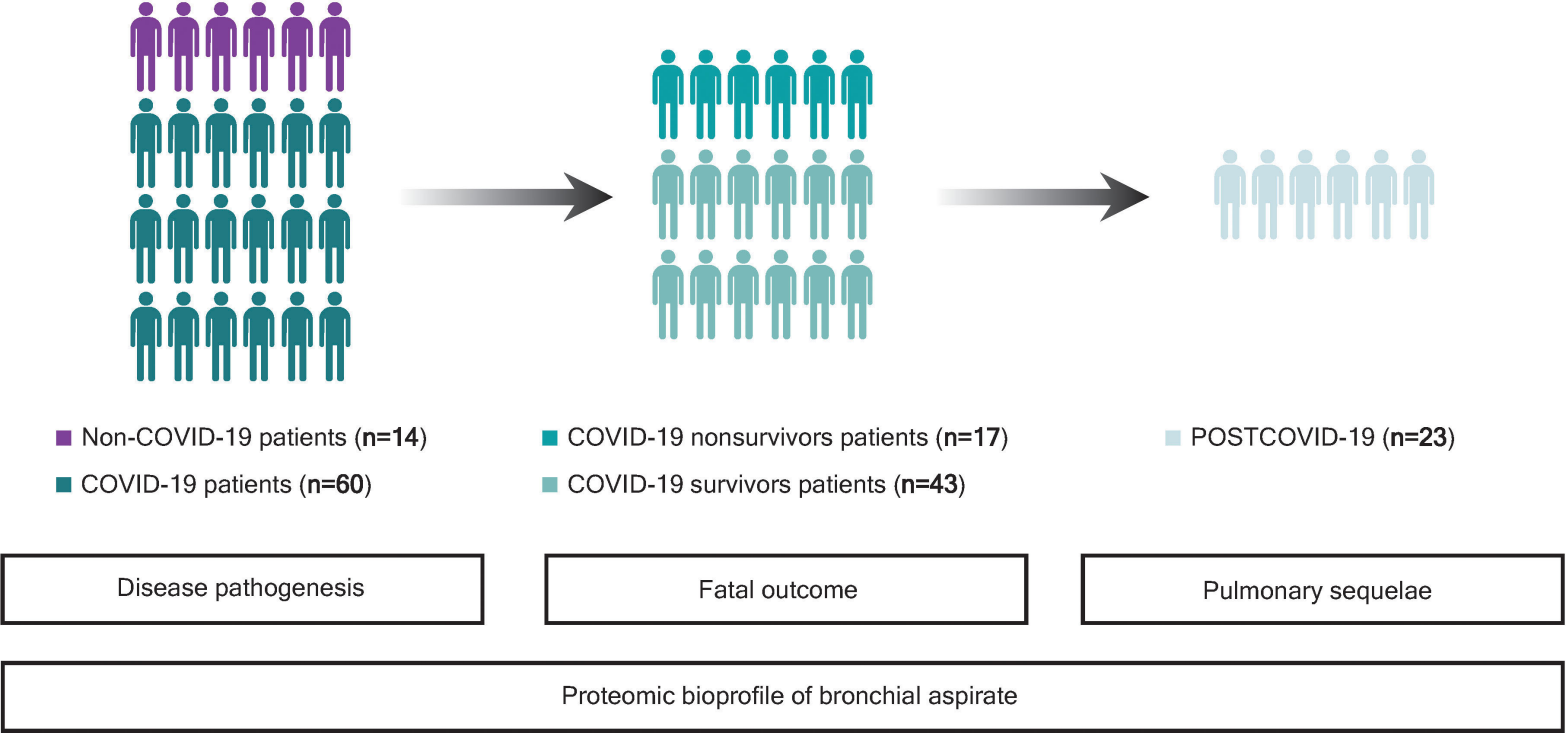
Study flowchart. First, bronchial aspirate (BAS) proteins associated with SARS-CoV-2 infection were evaluated in the whole study population, consisting of 14 critically ill non-COVID-19 and 60 COVID-19 patients, both groups assisted by IMV during their ICU stays (April-November 2020). For COVID-19 patients, BAS predictors of all-cause in-ICU mortality were analyzed in 17 nonsurvivors and 43 survivors. Then, the association between BAS proteins and functional and structural pulmonary sequelae was explored in 23 survivors of ICU stay who were subjected to a complete pulmonary evaluation 3 months after hospital discharge. Twenty survivors were unreachable or decided not to participate in follow-up (n = 17), were referred to another department (n = 2) or did not complete the pulmonary evaluation (n = 1).

This study was a multicenter and observational study. A summarized graphical description of the study design and patient population is depicted in **Figure 1**. The study population was composed of 74 critically ill COVID-19 and non-COVID-19 patients assisted by IMV during ICU stays from April 2020 to November 2020. The patients were 18 years old or older. COVID-19 patients presented a positive nasopharyngeal swab RT–qPCR test result for SARS-CoV-2 and developed ARDS based on the Berlin criteria. The main cause of ICU admission among non-COVID-19 patients was pneumonia (42.85%) (**Supplemental Table S1**). Samples were obtained at the Hospital Arnau de Vilanova (Lleida, Spain) with the support of IRBLleida Biobank (B.0000682) and “Plataforma Biobancos PT20/00021”. The procedure to obtain the samples was approved by the Research Ethics Committee (CEIC 2273). Samples were also provided by Biobank IdISBa B.0000527 (www.idisba.es) and CIBERES Pulmonary Biobank Consortium B.0000471, a network formed by eleven tertiary Spanish hospitals (www.ciberes.org) integrated into the Spanish National Biobanks Network (**Supplemental Table S2**). Samples were processed with the appropriate approval of the Ethics and Scientific Committees (s007-BBCOV) and with the collaboration of the health care services of the Hospital Universitario Son Espases and Hospital Son Llatzer (Palma, Spain). Twenty-three survivors of ICU stay were subjected to a complete pulmonary evaluation 3 months after hospital discharge, as previously described (17). The procedures included airway function [spirometry and diffusing capacity for carbon monoxide (D_LCO_)] and chest computed tomography (CT) examinations. The severity of pulmonary radiological abnormalities was evaluated using the total severity score (TSS), a marker of acute lung inflammation lesions (17). Comprehensive demographic, clinical and pharmacological data and information regarding hospital stay and outcomes were manually extracted from the electronic medical records by specialized clinical research assistants.

The study was performed in compliance with the ethical requirements of the Declaration of Helsinki. The participating patients or their relatives were informed about the research and provided their written informed consent before the use of their biological samples and clinical information.

### Outcomes

For acute phase analyses, the primary endpoint was all-cause in-ICU mortality. The secondary endpoints included the duration of IMV, ICU stay, and hospital stay after BAS collection. For postacute pulmonary sequelae, the primary endpoints were the D_LCO_ and TSS levels 3 months after hospital discharge.

### Bronchial aspirate collection and handling

BAS samples were obtained by bronchoscopy during the ICU stay and after IMV initiation using standardized procedures when clinically indicated, as previously described (13). The median [P25;P75] time from intubation to BAS collection was 7 [2;13] days. The samples were immediately aliquoted, frozen and stored at -80°C. The frozen aliquots from Biobank IdISBa B.0000527 and CIBERES Pulmonary Biobank Consortium B.0000471 were shipped on dry ice to the Lleida Institute for Biomedical Research (Lleida, Spain) and stored at the biobank.

### Bronchial aspirate protein profiling

BAS samples were pretreated following a SARS-CoV-2 heat inactivation protocol consisting of 15 minutes at 56°C, as described previously (18). Then, the samples were diluted to a concentration of 0.5 mg/mL in a final volume of 50 µL, stored at -80°C and shipped on dry ice. The detection and quantification of proteins were performed using the commercially available Proximity Extension Assay (PEA) methodology (Olink Bioscience, Uppsala, Sweden). The method exhibits high sensitivity, specificity and scalability (19). This technology uses a pair of oligonucleotide-labeled antibodies that bind to the corresponding target protein in a pairwise manner, thereby preventing cross-reactive events. Upon binding, the oligonucleotides hybridize, and a DNA polymerase extends and creates a unique sequence that can be detected by qPCR. Additional information is available at https://www.olink.com/resources-support/document-download-center. A preliminary study was performed to establish the optimal BAS dilution. A 1:4 dilution was selected based on detectability and the hook effect. Four Olink Target 96 panels were used: Immune Response, Inflammation, Metabolism and Organ Damage (**Supplemental Table S3**). These panels were selected based on the well-balanced inclusion of proteins implicated in the pathophysiology of the disease. Each panel included a total of 92 proteins. Three hundred sixty-four proteins were analyzed (four proteins were simultaneously measured in two panels). The samples were randomly distributed across the plates, and the quantification was performed with blinding of the study groups. The results are expressed in normalized protein expression units (NPX), which are relative log-transformed values. Proteins with ≥50% of samples less than the limit of detection (LOD) were excluded from further analysis. Two samples did not pass the quality control. These samples were removed from subsequent statistical analysis.

Additional quality control testing was performed correlating IL-6 levels, which were detected in overlapping assays. An optimal correlation was observed for COVID-19 and non-COVID-19 patients (rho=0.974) (**Supplemental Figure S1**).

### Statistical methods

The characteristics of the study populations were summarized by descriptive statistics. Data are presented as the medians [P25; P75] for continuous variables and as frequencies (percentage) for categorical variables. Continuous and categorical variables were compared between groups using the Mann-Whitney U test and Fisher’s exact test, respectively. Linear models with empirical Bayes statistic were used to evaluate differences in protein levels between groups (20). A propensity score (PS) was used for adjustment in the comparison between COVID-19 and non-COVID-19 patients. The PS was defined as the probability of COVID-19 and estimated using a logistic regression model where predictors were clinical and pharmacological data associated with COVID-19 and proteins levels (age, sex, obesity, chronic pulmonary disease, use of antibiotics, use of tocilizumab and use of corticoids). The survivor and nonsurvivor comparisons were adjusted by age. Principal component analysis (PCA) included the differentially detected proteins in univariate analyses. The correlation between continuous variables was calculated with Spearman’s rank correlation expressed using the coefficient rho. Volcano plots show the p value versus the fold change for differential expression or the p value versus the rho coefficient for correlations. The construction of protein signatures associated with the outcomes was performed by a selection process based on random forest (21, 22). Variable importance plots are displayed to illustrate the prediction value of each protein. In the all-cause in-ICU mortality substudy, the top five proteins with variable importance were used to construct a BAS protein-based prediction model. The model was based on predictions of a logistic model with all-cause in-ICU mortality as the outcome and the top five proteins as predictors. For individual proteins and the BAS protein-based prediction model, a cutoff point was established for fitted mortality risk using a maximally selected log-rank statistic (23). Kaplan-Meier curves were used to illustrate differences among groups in the time-to-event outcome, and the log-rank test was performed to assess statistical significance. The hazard ratio (HR) was estimated using Cox regression models including the dichotomized levels of the individual proteins and the BAS protein-based prediction model. Survival length was calculated from sample collection. In the postacute substudy, the relationship between the selected proteins and the outcomes was also evaluated using Generalized Additive Models (GAMs) with penalized cubic regression spline (24). The p value threshold defining statistical differential expression was set at <0.05. For postacute analysis, the association between proteins and lung parameters was considered biologically and clinically relevant when rho>0.3 and p value<0.1. Statistical analyses were performed using R software, version 4.0.2. To analyze lung cell-type specificities, we used single-cell RNA sequencing (scRNA-seq) data from the Genotype-Tissue Expression (GTEx) database (https://gtexportal.org/home/datasets) and evaluated the cell types compared to the proteins identified in univariate and multivariable analyses. Candidate FDA-approved drugs targeting proteins were identified using the Drug-Gene Interactions Database (25).

## Results

### Impact of SARS-CoV-2 infection on the proteomic bioprofile of bronchial aspirates from critically ill patients

First, we analyzed those factors associated with SARS-CoV-2 infection in patients under IMV. The baseline characteristics of the study population are depicted in **Table 1**. Compared to non-COVID-19 patients, COVID-19 patients presented a higher prevalence of obesity and a lower prevalence of chronic pulmonary diseases. At ICU admission, critically ill COVID-19 patients showed lower levels of PaO2, lymphocyte counts and monocyte counts. The duration of the ICU stay, use of non-IMV, and use of prone positioning were higher in COVID-19 patients. The use of antibiotics, tocilizumab and corticoids was also higher in COVID-19 patients.

**Table 1:** Characteristics of patients with non-COVID-19 and COVID-19 ARDS (study population 1).

	ALL	Non-COVID-19	COVID-19	p value	N
	*N=74*	*N=14*	*N=60*		
**Sociodemographic characteristics**					
Age (years)	66.0 [59.0;71.0]	66.5 [64.0;72.5]	64.5 [59.0;71.0]	0.243	74
Female	17 (23.0%)	6 (42.9%)	11 (18.3%)	0.075	74
Smoking history:				0.163	70
Current	10 (14.3%)	4 (30.8%)	6 (10.5%)		
Former	30 (42.9%)	4 (30.8%)	26 (45.6%)		
Nonsmoker	30 (42.9%)	5 (38.5%)	25 (43.9%)		
**Comorbidities**					
Hypertension	42 (56.8%)	7 (50.0%)	35 (58.3%)	0.789	74
Diabetes mellitus	29 (39.2%)	4 (28.6%)	25 (41.7%)	0.549	74
Obesity	36 (48.6%)	3 (21.4%)	33 (55.0%)	0.049	74
Cardiovascular disease	14 (18.9%)	3 (21.4%)	11 (18.3%)	0.721	74
Chronic lung disease	12 (16.2%)	7 (50.0%)	5 (8.33%)	0.001	74
Asthma	3 (4.05%)	1 (7.14%)	2 (3.33%)	0.472	74
Chronic kidney disease	6 (8.11%)	1 (7.14%)	5 (8.33%)	1.000	74
**ICU Admission**					
Oxygen saturation (%)	93.0 [88.0;95.2]	93.0 [92.0;93.5]	92.5 [88.0;95.2]	0.919	67
FiO_2_ (%)	70.0 [50.0;90.0]	50.0 [50.0;50.0]	70.0 [50.0;90.0]	0.350	53
PaO_2_ (mm Hg)	71.0 [55.8;96.2]	122 [84.5;140]	67.0 [52.8;83.0]	0.001	60
PaCO_2_ (mm Hg)	37.0 [32.0;43.0]	37.5 [34.0;44.0]	37.0 [31.5;41.4]	0.529	60
Glucose (mg/dL)	144 [115;207]	128 [110;174]	147 [116;215]	0.320	74
Creatinine (mg/dL)	0.90 [0.71;1.13]	0.83 [0.70;0.93]	0.94 [0.72;1.13]	0.423	73
C-reactive protein (mg/dL)	152 [86.0;224]	138 [70.0;252]	152 [86.0;216]	0.976	66
D-dimer (ng/mL)	397 [316;843]	475 [367;3883]	397 [301;843]	0.501	53
Leukocyte count (x10^9^/L)	8.02 [6.47;12.4]	11.0 [7.44;18.9]	7.78 [6.40;10.5]	0.087	74
Neutrophil count (x10^9^/L)	6.68 [5.28;10.2]	14.2 [5.48;18.8]	6.39 [5.27;8.55]	0.100	72
Lymphocyte count (x10^9^/L)	0.83 [0.52;1.33]	1.44 [1.05;1.87]	0.66 [0.51;1.17]	0.036	72
Monocyte count (x10^9^/L)	0.40 [0.21;0.62]	0.65 [0.50;0.97]	0.36 [0.21;0.54]	0.015	71
Platelet count (x10^9^/L)	230 [188;279]	218 [171;277]	232 [188;279]	0.571	74
**ICU Stay**					
ICU stay (days)	40.5 [16.2;58.0]	8.50 [6.25;18.2]	43.5 [30.5;61.5]	<0.001	74
Noninvasive mechanical ventilation	57 (78.1%)	7 (53.8%)	50 (83.3%)	0.030	73
Prone positioning	58 (81.7%)	2 (16.7%)	56 (94.9%)	<0.001	71
**Treatments**					
Antibiotics	64 (86.5%)	8 (57.1%)	56 (93.3%)	0.002	74
Hydroxychloroquine	23 (31.1%)	3 (21.4%)	20 (33.3%)	0.527	74
Tocilizumab	43 (58.1%)	2 (14.3%)	41 (68.3%)	0.001	74
Interferon beta	2 (2.70%)	0 (0.00%)	2 (3.33%)	1.000	74
Corticoids	59 (79.7%)	7 (50.0%)	52 (86.7%)	0.006	74
Remdesivir	13 (17.6%)	0 (0.00%)	13 (21.7%)	0.111	74

Continuous variables are expressed as the median [P25;P75] and categorical variables as n (%). FiO_2_, fraction of inspired oxygen; ICU, Intensive care unit; PaCO_2_, carbon dioxide partial pressure; PaO_2_, oxygen partial pressure.

**Figure 2:**
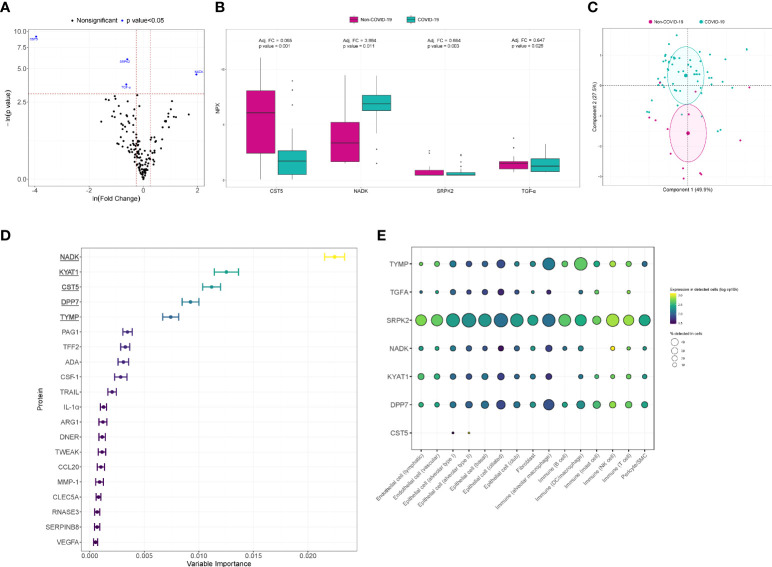
Proteomic bioprofiles in bronchial aspirates from COVID-19 and non-COVID-19 ARDS patients. **(A)** Volcano plot showing the p value versus the fold change. Each point represents a detected protein. Blue dots represent the detected proteins that showed significant differences considering a p value<0.05. The association was adjusted by a propensity score (PS) compound by age, sex, obesity, chronic pulmonary disease, use of antibiotics, use of tocilizumab and use of corticoids. **(B)** Boxplot including bronchial aspirate proteins that showed differences between study groups. The adjusted fold change is displayed, and the significance level for each comparison is described by the p value. **(C)** Principal component analysis. Each point represents a patient. **(D)** Variable importance plot displaying the most relevant proteins according to their contribution to the random forest model. **(E)** Cell enrichment analysis based on single-cell RNA-seq data from Genotype-Tissue Expression (GTex). Each row represents a protein, and each column represents a cell type. The color of the point shows the expression levels in detected cells, and the size of the point indicates the percentage of cells in which the expression was detected.

In the univariate analysis, four proteins were differentially detected after adjustment with PS (**Figures 2A, B**). CST5 (adjusted fold change (FC)=0.065, p value<0.001), SRPK2 (adjusted FC=0.664, p value=0.003) and TGF-α (adjusted FC=0.647, p value=0.026) showed lower levels in COVID-19 patients. NADK (adjusted FC=3.884, p value=0.011) levels were elevated in the same group of patients. A PCA including these significantly differentially detected proteins is displayed in **Figure 2C**. The four proteins allowed us to differentiate between individual COVID-19 and non-COVID-19 ARDS patients. When considering all of the detected proteins simultaneously, variable selection process based in random forest method identified NADK, KYAT1, CST5, DPP7 and TYMP as the top five proteins of variable importance (**Figure 2D**). Using available scRNA-seq datasets, we explored the expression in lung cells of the differentially detected proteins in univariate analysis and those identified in random forest analysis. The proteins were expressed in both immune and nonimmune lung cells (**Figure 2E**). The drug-gene interaction analysis identified several candidate drugs targeting TFG-α and TYMP (**Supplemental Table S4**).

### Specific proteomic factors quantified in bronchial aspirates predict all-cause in-ICU mortality and postacute pulmonary sequelae in critically ill patients with ARDS secondary to SARS-CoV-2 infection

Then, we evaluated the determinants associated with clinical outcomes during hospitalization and after hospital discharge in critically ill patients with SARS-CoV-2-induced ARDS. The sociodemographic, clinical, pharmacological and biochemical characteristics of the study population are presented in **Table 2**. The median age was 64.5 [59.0;71.0] years old, and 18.3% were women. No significant differences in sociodemographic, clinical, ventilatory or pharmacological variables were observed between survivors and nonsurvivors. As previously demonstrated in this study population (8), no great differences are observed between those patients with BAS samples and all patients under IMV or patients under IMV without BAS samples. Concerning the pulmonary sequelae, the main characteristics of the study population are reflected in **Table 3**. The median age was 62.0 [59.0;69.5] years old, and 21.7% were women. The time from hospital discharge to follow-up was 94.0 [85.8; 103] days. As expected, the study population presented a high prevalence of pulmonary diffusion impairment (D_LCO_ <80%): 95%. The median [P25;P75] TSS score was 10.5 [5.00;13.0].

**Table 2:** Characteristics of ICU COVID-19 survivors and nonsurvivors ARDS secondary to SARS-CoV-2 infection (study population 2).

	ALL	Survivors	Nonsurvivors	p value	N
	N=60	N=43	N=17		
**Sociodemographic characteristics**					
Age (years)	64.5 [59.0;71.0]	63.0 [57.0;70.0]	67.0 [63.0;71.0]	0.123	60
Female	11 (18.3%)	8 (18.6%)	3 (17.6%)	1.000	60
Smoking history:				0.481	57
Current	6 (10.5%)	3 (7.32%)	3 (18.8%)		
Former	26 (45.6%)	19 (46.3%)	7 (43.8%)		
Nonsmoker	25 (43.9%)	19 (46.3%)	6 (37.5%)		
**Comorbidities**					
Hypertension	35 (58.3%)	26 (60.5%)	9 (52.9%)	0.809	60
Diabetes mellitus	25 (41.7%)	20 (46.5%)	5 (29.4%)	0.358	60
Obesity	33 (55.0%)	23 (53.5%)	10 (58.8%)	0.931	60
Cardiovascular disease	11 (18.3%)	7 (16.3%)	4 (23.5%)	0.712	60
Chronic lung disease	5 (8.33%)	3 (6.98%)	2 (11.8%)	0.616	60
Asthma	2 (3.33%)	2 (4.65%)	0 (0.00%)	1.000	60
Chronic kidney disease	5 (8.33%)	4 (9.30%)	1 (5.88%)	1.000	60
**ICU admission**					
Oxygen saturation (%)	92.5 [88.0;95.2]	92.0 [88.0;94.8]	94.1 [91.0;96.0]	0.114	58
FiO_2_ (%)	70.0 [50.0;90.0]	60.0 [48.8;82.5]	75.0 [60.0;90.0]	0.202	48
PaO_2_ (mm Hg)	67.0 [52.8;83.0]	65.0 [51.5;80.0]	76.0 [56.0;83.0]	0.501	48
PaCO_2_ (mm Hg)	37.0 [31.5;41.4]	38.0 [34.0;41.6]	32.0 [29.0;40.0]	0.285	48
Glucose (mg/dL)	147 [116;215]	164 [116;235]	129 [121;156]	0.082	60
Creatinine (mg/dL)	0.94 [0.72;1.13]	0.93 [0.72;1.10]	1.00 [0.69;1.13]	0.857	60
C-reactive protein (mg/L)	152 [86.9;216]	151 [86.9;192]	161 [106;289]	0.330	58
D-dimer (ng/mL)	397 [301;843]	422 [323;1388]	367 [285;432]	0.138	49
Leukocyte count (x10^9^/L)	7.78 [6.40;10.5]	7.39 [6.28;9.94]	8.61 [6.93;14.1]	0.147	60
Neutrophil count (x10^9^/L)	6.39 [5.27;8.55]	6.32 [5.35;8.27]	7.01 [4.92;9.41]	0.604	59
Lymphocyte count (x10^9^/L)	0.66 [0.51;1.17]	0.64 [0.51;1.09]	0.73 [0.51;2.14]	0.273	59
Monocyte count (x10^9^/L)	0.36 [0.21;0.54]	0.34 [0.21;0.48]	0.48 [0.21;0.56]	0.203	59
Platelet count (x10^9^/L)	232 [188;279]	237 [202;276]	203 [139;287]	0.106	60
**ICU Stay**					
ICU stay (days)	43.5 [30.5;61.5]	44.0 [25.5;62.0]	41.0 [31.0;51.0]	0.670	60
Noninvasive mechanical ventilation	50 (83.3%)	37 (86.0%)	13 (76.5%)	0.448	60
Prone positioning	56 (94.9%)	39 (92.9%)	17 (100%)	0.550	59
**Treatments**					
Antibiotics	56 (93.3%)	40 (93.0%)	16 (94.1%)	1.000	60
Hydroxychloroquine	20 (33.3%)	14 (32.6%)	6 (35.3%)	1.000	60
Tocilizumab	41 (68.3%)	30 (69.8%)	11 (64.7%)	0.943	60
Interferon beta	2 (3.33%)	2 (4.65%)	0 (0.00%)	1.000	60
Corticoids	52 (86.7%)	36 (83.7%)	16 (94.1%)	0.420	60
Remdesivir	13 (21.7%)	8 (18.6%)	5 (29.4%)	0.488	60

Continuous variables are expressed as the median [P25; P75] and categorical variables as n (%). FiO_2_, fraction of inspired oxygen; ICU, Intensive care unit; PaCO_2_, carbon dioxide partial pressure; PaO_2_, oxygen partial pressure.

**Table 3:** Characteristics of post-Covid patients (study population 3).

Sociodemographic characteristics		
Age (years)	62.0 [59.0;69.5]	23
Female	5 (21.7%)	23
Smoking history:		22
Current	1 (4.55%)	
Former	12 (54.5%)	
Nonsmoker	9 (40.9%)	
**Comorbidities**		
Hypertension	15 (65.2%)	23
Diabetes mellitus	12 (52.2%)	23
Obesity	12 (52.2%)	23
Cardiovascular disease	3 (13.0%)	23
Chronic lung disease (negative)	23 (100%)	23
Asthma	1 (4.35%)	23
Chronic kidney disease	2 (8.70%)	23
**ICU admission**		
Oxygen saturation (%)	94.0 [88.2;95.3]	22
FiO_2_ (%)	65.0 [52.5;80.0]	22
PaO_2_ (mm Hg)	62.0 [54.0;71.0]	17
PaCO_2_ (mm Hg)	38.0 [35.0;41.0]	17
Glucose (mg/dL)	190 [120;252]	23
Creatinine (mg/dL)	0.86 [0.72;1.21]	23
C-reactive protein (mg/L)	151 [86.9;179]	22
D-dimer (ng/mL)	389 [301;811]	21
Leukocyte count (x10^9^/L)	7.32 [6.28;8.32]	23
Neutrophil count (x10^9^/L)	5.99 [5.15;7.16]	23
Lymphocyte count (x10^9^/L)	0.79 [0.54;1.06]	23
Monocyte count (x10^9^/L)	0.29 [0.21;0.38]	23
Platelet count (x10^9^/L)	231 [194;275]	23
**ICU stay**		
ICU stay (days)	44.0 [31.0;62.0]	23
Noninvasive mechanical ventilation	20 (87.0%)	23
Prone positioning	21 (91.3%)	23
**Treatments**		
Antibiotics	22 (95.7%)	23
Hydroxychloroquine	3 (13.0%)	23
Tocilizumab	20 (87.0%)	23
Interferon beta	1 (4.35%)	23
Corticoids	22 (95.7%)	23
Remdesivir	5 (21.7%)	23
**Post-COVID parameters**		
D_LCO_	49.9 [41.5;62.3]	20
<60%	13 (65.0%)	
<80%	6 (30.0%)	
≥80%	1 (5.00%)	
TSS	10.5 [5.00;13.0]	22

Continuous variables are expressed as the median [P25;P75] and categorical variables as n (%). D_LCO_, carbon monoxide diffusing capacity; FiO_2_, fraction of inspired oxygen; ICU, Intensive care unit; PaCO_2_, carbon dioxide partial pressure; PaO_2_, oxygen partial pressure; TSS, total severity score.

**Figure 3:**
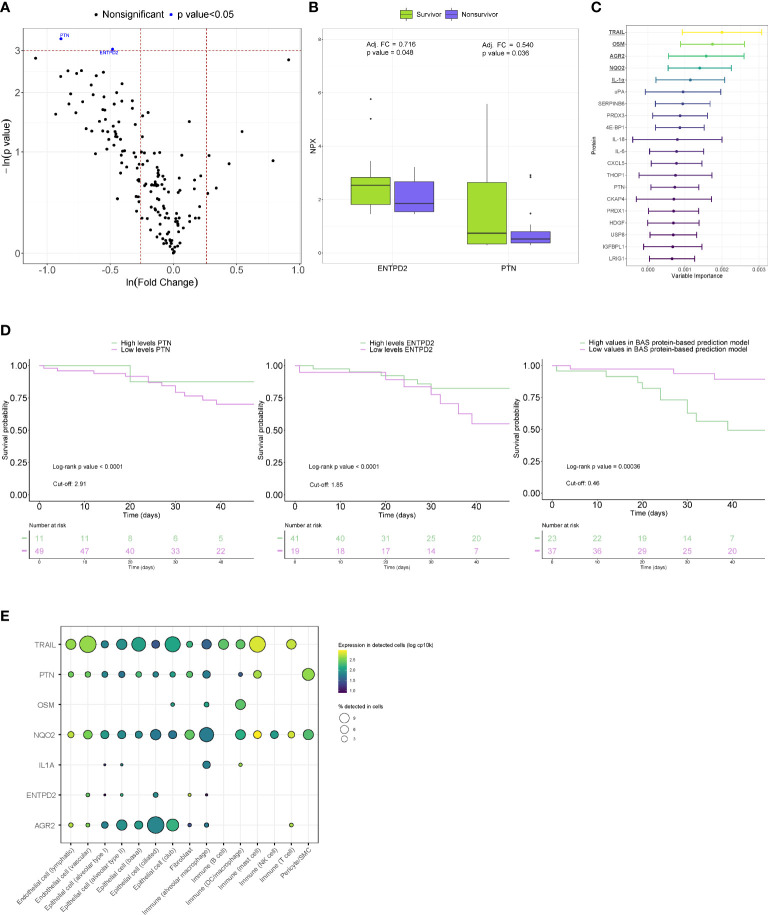
Proteomic bioprofiles of bronchial aspirates from survivors and nonsurvivors of ARDS secondary to SARS-CoV-2 infection. **(A)** Volcano plot showing the p value versus the fold change. Each point represents a detected protein. Blue dots represent the detected proteins that showed significant differences considering a p value<0.05. The association was adjusted by age. **(B)** Boxplot including bronchial aspirate proteins that showed differences between study groups. The adjusted fold change is displayed, and the significance level for each comparison is described by the p value. **(C)** Variable importance plot displaying the most relevant proteins according to their contribution to the random forest model. **(D)** Kaplan-Meier estimations for the individual proteins and BAS protein-based prediction model. Log-rank p values are displayed. **(E)** Cell enrichment analysis based on single-cell RNA-seq data from Genotype-Tissue Expression (GTex). Each row represents a protein, and each column represents a cell type. The color of the point shows the expression levels in detected cells, and the size of the point indicates the percentage of cells in which the expression was detected.

When proteins were analyzed individually, concomitant reductions in PTN (adjusted FC=0.540, p value=0.036) and ENTPD2 (adjusted FC =0.716, p value=0.048) were observed in fatal cases after adjusting for age (**Figures 3A, B**). In non-COVID-19 patients, similar levels between survivors and nonsurvivors of ICU stay were observed for both proteins (**Supplemental Figure S2**). In the random forest statistical model, TRAIL, OSM, AGR2, NQO2 and IL-1α were the five most important proteins for all-cause in-ICU mortality prediction (**Figure 3C**). We next tested the capacity of PTN and ENTPD2 and a BAS protein-based prediction model composed of the proteins with the strongest variable importance to predict the risk of in-ICU mortality at the time of BAS collection. Kaplan-Meier curves revealed a clear separation of the risk of in-ICU mortality between the low and high PTN and ENTPD2 subgroups (**Figure 3D**). Patients with lower values of PTN and ENTPD2 showed a higher in-ICU mortality risk, with HRs of 4.00 and 2.14, respectively. Separation of survival between patients with different levels in the BAS protein-based prediction model was also observed (**Figure 3D**). Indeed, higher levels of the model were directly associated with higher mortality risk: HR=6.25. Analysis of data from the GTEx project showed a variable expression pattern in lung epithelial, immune and endothelial cells (**Figure 3E**). We identified FDA-approved drugs that targets NQO2 and IL-1α (**Supplemental Table S5**). Among the secondary endpoints, no correlations were observed between the protein levels and days under IMV, duration of ICU stay, or duration of hospital stay (**Supplemental Table S5**).

**Figure 4:**
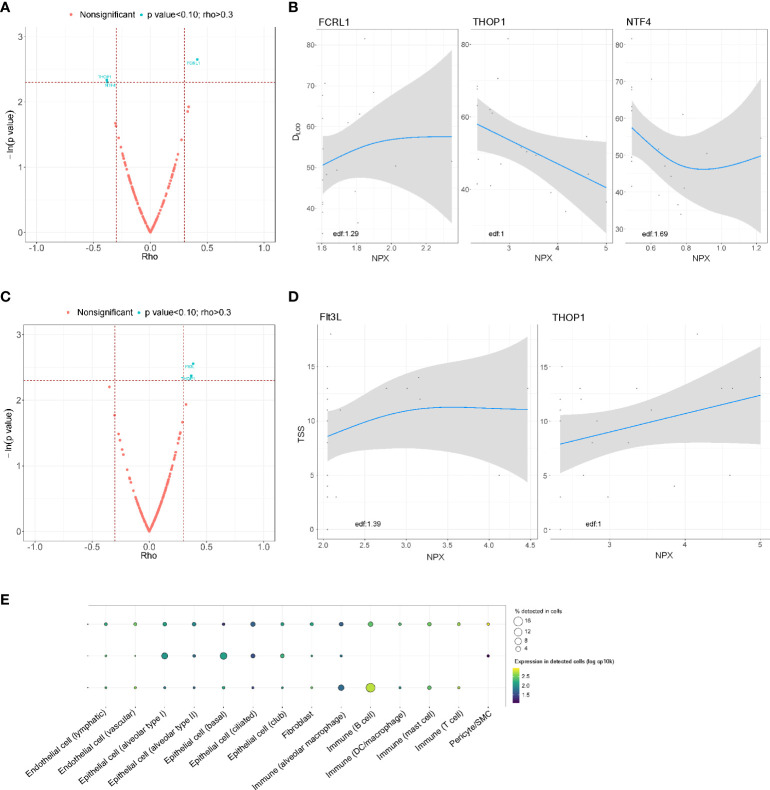
Association between the proteomic bioprofile in bronchial aspirates with diffusion capacity and structural features in survivors of ARDS secondary to SARS-CoV-2 infection. **(A)** Volcano plot showing the p value versus the rho coefficient for each detected protein after comparison of D_LCO_ levels. Each dot represents a protein. Blue dots represent proteins that showed a biologically and clinically relevant correlation with D_LCO_ levels; **(B)** GAM modeling for D_LCO_ and the levels of each of the selected proteins; **(C)** Volcano plot showing the p value versus the rho coefficient for each detected protein after comparison of TSS levels. Each dot represents a protein. Blue dots represent proteins that showed a biologically and clinically relevant correlation with TSS levels; **(D)** GAM modeling for TSS and the levels of each of the selected proteins. **(E)** Cell enrichment analysis based on single-cell RNA-seq data from Genotype-Tissue Expression (GTex). Each row represents a protein, and each column represents a cell type. The color of the point shows the expression levels in detected cells, and the size of the point indicates the percentage of cells in which the expression was detected. Edf: estimated degrees of freedom.

In the postacute substudy, FCRL1 (rho = 0.413) was positively correlated with lung function 3 months after hospital discharge, whereas THOP1 (rho = -0.382) and NTF4 (rho = -0.379) showed an inverse correlation with D_LCO_ (**Figures 4A, B**). A positive correlation of TSS with proteins Flt3L (rho=0.384) and THOP1 (rho = 0.367) was also observed (**Figures 4C, D**). In the GAM, a linear relationship was observed for THOP1 for both parameters. Except for Flt3L, all BAS proteins were detected in lung-resident cells (**Figure 4E**). No approved-FDA drugs were found in the *in silico* analysis.

## Discussion

In the present study, we provide compelling evidence for the potential use of proteomics in BAS samples for molecularly phenotyping patients with SARS-CoV-2-induced ARDS. We report three major findings: i) SARS-CoV-2 infection induces significant alterations in the protein pattern of BAS; ii) specific protein factors quantified in BAS samples constitute novel biomarkers for risk stratification of fatal outcomes and postacute pulmonary sequelae; and iii) the protein fingerprint of BAS extends the current knowledge on the nature of the biological response to SARS-CoV-2 infection in the lower respiratory tract.

### Proteomic determinants of COVID-19 pathology and adverse outcomes in critically ill patients

The cysteine proteinase inhibitor CST5 was the protein that showed the greatest downregulation in COVID-19. CST5, similar to other members of the cystatin superfamily (26), has been described as part of the nonimmune protective system due to its role in counteracting the harmful effects of proteinases from pathogens, including viruses. Collins and Grubb (27) demonstrated that recombinant human CST5 inhibits human coronavirus replication and slows the release of the virus from infected lung cells. CST5 has also been reported to be an inhibitor of herpes simplex virus-induced apoptosis (28). A possible relationship between the low levels of CST5 and SARS-CoV-2 infectivity should not be discarded. Our results support the recently described protease-anti-protease imbalance in the airways of SARS-CoV-2-ARDS patients (29). Other proteins detected in the univariate and multivariable analyses could be indicators of host responses to SARS-CoV-2 infection. KYAT1, previously linked to SARS-CoV-2 infection (30), is a regulator of kynurenine metabolism, a pathway implicated in inflammation and immunity (31). The protein that showed the greatest variable importance, NADK, governs NADP biosynthesis, which plays an essential role in antioxidant defense and metabolism (32). The plasma concentration of TYMP has been proposed to be an acuity marker for COVID-19 diagnosis and has been associated with inflammation and organ damage (33), hallmarks of the disease (34, 35).

Although corticosteroids and antiviral drugs have demonstrated efficacy in the treatment of the infection, efficient therapeutic strategies in critical patients are currently lacking. Understanding the underlying mechanisms that mediate clinical decompensation in severe clinical courses is a key requirement to design novel pharmacological agents. Here, the comparison of fatal cases to survivors suggests a poor reparative response as a major pathway associated with death. PTN showed profound downregulation in nonsurvivors of the disease. This protein is a multifunctional trophic factor that participates in cell differentiation, proliferation and growth (36). PTN expression is elevated in response to injury in different organs and has been associated with tissue repair by promoting regeneration and angiogenesis (37). PTN has been previously proposed as an extracellular mediator involved in lung epithelial repair. Weng et al. (38) reported that PTN promotes wound healing of fetal alveolar epithelial type II cells *in vitro* in a dose-dependent manner. The reparative response is blocked when an anti-PTN antibody is added to the medium. The role of PTN in these mechanisms is supported by independent investigations that proposed that PTN signaling mediates neovascular formation after acute ischemic brain injury (39). Survival of the infection depends on the balance between pathogen clearance and the maintenance of lung function. Therefore, a causal association between the inability to organize a productive reparative response and the increased risk of death in critically ill patients should not be excluded. Future studies should explore this hypothesis.

Our proteomic analysis provided evidence on the potential biological pathways mediating the lung sequelae of survivors to severe clinical courses of the disease. Different BAS proteins were correlated with D_LCO_ and TSS 3 months after hospital discharge. THOP1 showed a dose-response relationship with both functional and structural lung characteristics. How this protein participates in the development of lung sequelae or recovery is unclear. Previous evidence suggested a role of THOP1 in the metabolism of different biologically functional peptides implicated in mechanisms such as cell proliferation and differentiation and angiogenesis (40). A role in the regulation of energy metabolism and type 2 diabetes, recently reported as a risk factor for postacute COVID-19 sequelae (41), has also been proposed (42). Regarding immune function, THOP1 strengthens the immune defense against intracellular pathogens in cytotoxic T lymphocytes (43). Similarly, Flt3L and FCRL1 have been implicated in the regulation of immune cell development and activation and lung fibrosis (44, 45). All of these factors are in line with the multifactorial nature of postintensive care syndrome pathophysiology (46, 47). Whether the alteration of these pathways during the acute phase mediates resolution and repair merits further investigation. Interestingly, although our drug-gene interaction analysis proposed several candidate drugs that can target proteins associated with the COVID-19 pathology and its fatal outcomes, no FDA-approved drugs were identified for postacute sequelae. Therefore, current findings may be useful to develop novel therapeutic interventions.

### Predictors of all-cause in-ICU mortality and postacute pulmonary sequelae in critically ill patients with ARDS secondary to SARS-CoV-2 infection

The group of patients with SARS-CoV-2-induced ARDS under IMV presents the highest morbidity and mortality rates. However, tools to assist in risk stratification are lacking. Since the lung compartmentalization of specific biomarkers has been described in the context of ARDS, and the proteome signature differs in the lung and blood compartments (48), assessing lung-borne proteins is crucial for the development of tools for the management of pulmonary disease. In this multicentric study that analyzed several hundred BAS proteins from patients under invasive ventilatory support, we found that two individual proteins, PTN and ENTPD2, were predictors of fatal outcomes. Due to the anticipated heterogeneity of a complex disease such as ARDS, we incorporated additional approaches for proteomic modeling. Using random forest models, we defined a BAS protein-based model composed of the most important predictors of all-cause in-ICU mortality: TRAIL, OSM, AGR2, NQO2 and IL-1α. Furthermore, we identified individual proteins that, when quantified in BAS samples after IMV initiation and during the ICU stay, might predict pulmonary function (FCRL1, THOP1 and NTF4) and radiological features (Flt3L and THOP1) three months after hospital discharge. Comparison of our findings with those of other authors is hampered by the paucity of studies that have evaluated the proteomic bioprofiles of BAS samples. Divergences due to the type of sample analyzed, lung region explored, and the detection method used should be expected. Nevertheless, the results support previous proteomic studies in different matrices (49, 50).

Using a standardized, high-throughput and high-sensitivity technique, we demonstrate the feasibility and usefulness of BAS proteomics in patients under IMV. BAS-based protein testing constitutes an innovative tool for the development of risk algorithms for fatal outcomes in the ICU, which ultimately could facilitate clinical management and the selection of patients for clinical trials. This use is also applicable in survivors at high risk for developing lung sequelae. Measuring BAS proteins for the early prediction of lung sequelae could be useful to implement specific strategies for patient care, monitoring and treatment after ICU-hospital discharge. The clinical interest is relevant since quantifiable risk factors for postacute sequelae are poorly described. Further studies are required to evaluate the impact of current findings on patient management and decision making.

### Strengths and limitations of the study

The strengths include the multicenter character of the study, the use of a sensitive and specific proteomic method and the strict quality control of the clinical and protein data. The study integrated novel proteomic approaches and standard clinical care. The safety of BAS collection in comparison with other respiratory samples of the lower respiratory tract should be emphasized. Nevertheless, this study has some limitations. First, although the sample size was similar to that in other proteomic studies (51), the cohort is not sufficiently large to analyze the specific cause of mortality or the improvement of a clinical model. The small sample size of the non-COVID-19 ARDS should be highlighted. Second, confounding factors due to comorbidities, clinical management and inpatient complications should be considered. These limitations warrant further studies in large-scale cohorts, which is beyond the scope of the current investigation. Third, the causes of ICU admission of non-COVID-19 patients should be more homogeneous and preferably limited to respiratory infection. Forth, the association between the proteomic bioprofiles in respiratory samples of non-COVID-19 patients admitted to the ICU to their follow-ups would provide valuable information. Unfortunately, information related to postacute pulmonary sequelae was unavailable for this study group. Fifth, although our findings support mechanistic hypotheses, causality cannot be ascertained. Sixth, BAS has remarkable complexity and could be compounded by immune, pulmonary, epithelial or vascular cells, and it is not possible to precisely define the protein origin. Seventh, the utilized proteomic technology yields semiquantified concentrations. Eighth, measured proteins were the results of a previous selection of predesigned Olink panels.

## Conclusions

The BAS proteome associated with ARDS secondary to SARS-CoV-2 emerges as a novel source of information on the mechanistic pathways implicated in the host response and its adverse outcomes. The deregulated proteins, individually or collectively, constitute novel therapeutic candidates and biomarkers to predict the evolution and sequelae of SARS-CoV-2-induced ARDS in patients under IMV. The current findings provide a comprehensive knowledge map for further investigations.

## Data availability statement

The original contributions presented in the study are included in the article/**Supplementary Material**. Further inquiries can be directed to the corresponding author.

## Ethics statement

The studies involving human participants were reviewed and approved by Ethics and Scientific Committees (s007-BBCOV)/Research Ethics Committee (CEIC 2273). The patients/participants provided their written informed consent to participate in this study.

## Author contributions

MM, IB, FB, and DG-C contributed to the study concept and design. MM, SG, JV, JG, DP, CG-P, AM-M, MG-H, MP-P, TB, GT, JC, CB, JA, LS, AC, LF-B, RF, DG-G, JL-B, RM, AM, OP, JR, AT, and FB contributed to the data acquisition. MM, IB, FB, and DG-C contributed to the data analysis and interpretation. All authors contributed to the manuscript draft, critically revised the manuscript for important intellectual content and approved the final version. DG-C is the guarantor of the paper.

## Funding

MM is the recipient of a predoctoral fellowship (PFIS: FI21/00187) from Instituto de Salud Carlos III. MG-H is the recipient of a predoctoral fellowship from the University of Lleida. DG-C has received financial support from Instituto de Salud Carlos III (Miguel Servet 2020: CP20/00041), co-funded by the European Social Fund (ESF)/”Investing in your future”. Financial support was provided by the Instituto de Salud Carlos III de Madrid (COV20/00110), co-funded by the European Development Regional Fund (A Way to Achieve Europe program) and Centro de Investigación Biomedica En Red – Enfermedades Respiratorias (CIBERES). CIBERES is an initiative of the Instituto de Salud Carlos III. We were further supported by: Programa de donaciones “estar preparados”; UNESPA (Madrid, Spain); and Fundación Francisco Soria Melguizo (Madrid, Spain). AC is supported by Instituto de Salud Carlos III (Sara Borrell 2021: CD21/00087).

## Acknowledgments

The authors are indebted to María Arguimbau, Raquel Campo, Natalia Jarillo, Javier Muñoz, Elisabeth Sancho, Fernando Gómez and Manuel Sánchez for their extensive support with project management and article preparation. We particularly want to acknowledge the patients, Biobank IdISBa and CIBERES Pulmonary Biobank Consortium (PT17/0015/0001), a member of the Spanish National Biobanks Network financed by the Carlos III Health Institute, and the Units of Intensive Care, Clinical Analysis and Pulmonology of Hospital Universitario Son Espases and Hospital Son Llatzer for their collaboration. This work was supported by IRBLleida Biobank (B.0000682) and “Plataforma Biobancos PT20/00021”. The human sample manipulation was performed in the Cell Culture Technical Scientific Service of the Universitat de Lleida (Lleida, Catalonia, Spain).

## Conflict of interest

The authors declare that the research was conducted in the absence of any commercial or financial relationships that could be construed as a potential conflict of interest.

## Publisher’s note

All claims expressed in this article are solely those of the authors and do not necessarily represent those of their affiliated organizations, or those of the publisher, the editors and the reviewers. Any product that may be evaluated in this article, or claim that may be made by its manufacturer, is not guaranteed or endorsed by the publisher.
