# Supplementary material for: Multiplex protein profiling of bronchial aspirates reveals disease-, mortality- and respiratory sequelae-associated signatures in critically ill patients with ARDS secondary to SARS-CoV-2 infection
Source: Front Immunol. 2022 Jul 29;13:942443. doi: 10.3389/fimmu.2022.942443 (PMC9373836; doi:10.3389/fimmu.2022.942443)
Supplement: Supplementary file 1 [file DataSheet_1.docx]

Supplementary Material

Multiplex protein profiling of bronchial aspirates reveals disease-, mortality- and respiratory sequelae-associated signatures in critically ill patients with ARDS secondary to SARS-CoV-2 infection

**Marta Molinero,^1^ Silvia Gómez,^1,2^ Iván D. Benítez,^1,2^ J. J. Vengoechea,^1,2^ Jessica González,^1,2^ Dinora Polanco,^1^ Clara Gort-Paniello,^1,2^ Anna Moncusí-Moix,^1,2^ María C. García-Hidalgo,^1^ Manel Perez-Pons,^1,2^ Thalía Belmonte,^1,2^ Gerard Torres,^1,2^ Jesús Caballero,^3^ Carme Barberà,^4^ Jose Ignacio Ayestarán Rota,^5^ Lorenzo Socías Crespí,^6^ Adrián Ceccato,^2^ Laia Fernández-Barat,^2,7^ Ricard Ferrer,^2,8^ Dario Garcia-Gasulla,^9^ Jose Ángel Lorente-Balanza,^2,10^ Rosario Menéndez,^2,11^ Ana Motos,^2,7^ Oscar Peñuelas,^2,10^ Jordi Riera,^2,8^ Antoni Torres,^2,7^ Ferran Barbé,^1,2^ David de Gonzalo-Calvo,^1,2,*^**

on behalf of the CIBERESUCICOVID Project (COV20/00110, ISCIII).

^1^ Translational Research in Respiratory Medicine, University Hospital Arnau de Vilanova and Santa Maria, IRBLleida, Lleida, Spain.

^2^ CIBER of Respiratory Diseases (CIBERES), Institute of Health Carlos III, Madrid, Spain.

^3^ Intensive Care Department, University Hospital Arnau de Vilanova, IRBLleida, Lleida, Spain.

^4^ Intensive Care Department, University Hospital Santa María, IRBLleida, Lleida, Spain.

^5^ Intensive Care Unit, Son Espases University Hospital, Palma de Mallorca, Spain.

^6^ Critical Care Department, Son Llàtzer Hospital, Palma de Mallorca, Spain.

^7^ Servei de Pneumologia, Hospital Clinic; Universitat de Barcelona; IDIBAPS, Barcelona, Spain.

^8^ Intensive Care Department, Vall d’Hebron Hospital Universitari. SODIR Research Group, Vall d’Hebron Institut de Recerca (VHIR), Spain.

^9^ Barcelona Supercomputing Center (BSC), Barcelona, Spain.

^10^ Hospital Universitario de Getafe, Madrid, Spain.

^11^ Pulmonology Service, University and Polytechnic Hospital La Fe, Valencia, Spain.

*** Correspondence:**

David de Gonzalo-Calvo, Ph.D.

Translational Research in Respiratory Medicine, University Hospital Arnau de Vilanova and Santa Maria, IRBLleida.

Avda. Alcalde Rovira Roure 80 · 25198 Lleida, Spain.

E-mail: [dgonzalo@irblleida.cat](mailto:dgonzalo@irblleida.cat)

# Supplementary Tables

**Supplemental Table S1**. Causes of ICU admission among non-COVID-19 patients.

| **Disease** | **Patients** |
| --- | --- |
| Pneumonia | 6 (42.85%) |
| Brain edema | 2 (14.28%) |
| Hemoptysis | 1 (7.14%) |
| Cervical abscess (surgical intervention) | 1 (7.14%) |
| Severe myasthenia | 1 (7.14%) |
| Acute peritonitis | 1 (7.14%) |
| Cardiorespiratory arrest | 1 (7.14%) |
| Cardiogenic shock | 1 (7.14%) |

**Supplemental Table S2. Hospitals included in the CIBERES Pulmonary Biobank Consortium.**

| Hospital Universitario 12 de Octubre |
| --- |
| Hospital Clínic de Barcelona |
| Hospital Universitario de Getafe |
| Hospital Universitario de Bellvitge |
| Hospital del Mar |
| Fundación Jiménez Díaz Capio |
| Hospital Universitario Son Espases |
| Hospital Joan XXIII de Tarragona |
| Hospital Universitario Germans Trias i Pujol |
| Hospital Universitario Vall d'Hebrón |
| Consorcio Hospital General Universitario de Valencia |

**Supplemental Table S3.**  Proteins included in current investigation.

| **Immune Response Panel** | | | |
| --- | --- | --- | --- |
| *Target* | *UniProt number* | *Target* | *UniProt number* |
| Allergin-1 (MILR1) | Q7Z6M3 | Interleukin-10 (IL10) | P22301 |
| Amphiregulin (AR) (AREG) | P15514 | Interleukin-12 receptor subunit beta-1 (IL12RB1) | P42701 |
| Aryl hydrocarbon receptor nuclear translocator (ARNT) | P27540 | Islet cell autoantigen 1 (ICA1) | Q05084 |
| Baculoviral IAP repeat-containing protein 2 (BIRC2) | Q13490 | Keratin, type I cytoskeletal 19 (KRT19) | P08727 |
| Beta-galactosidase (GLB1) | P16278 | Leukocyte immunoglobulin-like receptor subfamily B member 4 (LILRB4) | Q8NHJ6 |
| Butyrophilin subfamily 3 member A2 (BTN3A2) | P78410 | Lymphocyte activation gene 3 protein (LAG3) | P18627 |
| CD83 antigen (CD83) | Q01151 | Lymphocyte antigen 75 (LY75) | O60449 |
| Contactin-associated protein-like 2 (CNTNAP2) | Q9UHC6 | Lysosome-associated membrane glycoprotein 3 (LAMP3) | Q9UQV4 |
| Corneodesmosin (CDSN) | Q15517 | Mannan-binding lectin serine protease 1 (MASP1) | P48740 |
| Corticosteroid 11-beta-dehydrogenase isozyme 1 (HSD11B1) | P28845 | Merlin (NF2) | P35240 |
| Coxsackievirus and adenovirus receptor (CXADR) | P78310 | Methylated-DNA--protein-cysteine methyltransferase (MGMT) | P16455 |
| C-type lectin domain family 4 member A (CLEC4A) | Q9UMR7 | Natural cytotoxicity triggering receptor 1 (NCR1) | O76036 |
| C-type lectin domain family 4 member C (CLEC4C) | Q8WTT0 | Natural killer cells antigen CD94 (KLRD1) | Q13241 |
| C-type lectin domain family 4 member D (CLEC4D) | Q8WXI8 | Neurabin-2 (PPP1R9B) | Q96SB3 |
| C-type lectin domain family 4 member G (CLEC4G) | Q6UXB4 | Neurotrophin-4 (NTF4) | P34130 |
| C-type lectin domain family 6 member A (CLEC6A) | Q6EIG7 | Nuclear factor of activated T-cells, cytoplasmic 3 (NFATC3) | Q12968 |
| C-type lectin domain family 7 member A (CLEC7A) | Q9BXN2 | Parathyroid hormone/parathyroid hormone-related peptide receptor (PTH1R) | Q03431 |
| Cytoskeleton-associated protein 4 (CKAP4) | Q07065 | PC4 and SFRS1-interacting protein (PSIP1) | O75475 |
| Diacylglycerol kinase zeta (DGKZ) | Q13574 | Peroxiredoxin-1 (PRDX1) | Q06830 |
| Discoidin, CUB and LCCL domain-containing protein 2 (DCBLD2) | Q96PD2 | Peroxiredoxin-5, mitochondrial (PRDX5) | P30044 |
| DNA fragmentation factor subunit alpha (DFFA) | O00273 | Phosphoinositide 3-kinase adapter protein 1 (PIK3AP1) | Q6ZUJ8 |
| Dual adapter for phosphotyrosine and 3-phosphotyrosine and 3-phosphoinositide (DAPP1) | Q9UN19 | Plexin-A4 (PLXNA4) | Q9HCM2 |
| Dynactin subunit 1 (DCTN1) | Q14203 | Polypeptide N-acetylgalactosaminyltransferase 3 (GALNT3) | Q14435 |
| E3 ubiquitin-protein ligase TRIM21 (TRIM21) | P19474 | Probable ATP-dependent RNA helicase DDX58 (DDX58) | O95786 |
| Egl nine homolog 1 (EGLN1) | Q9GZT9 | Protein FAM3B (FAM3B) | P58499 |
| Eotaxin (CCL11) | P51671 | Protein HEXIM1 (HEXIM1) | O94992 |
| Eukaryotic translation initiation factor 4 gamma 1 (EIF4G1) | Q04637 | Protein kinase C theta type (PRKCQ) | Q04759 |
| Eukaryotic translation initiation factor 5A-1 (EIF5A) | P63241 | Protein sprouty homolog 2 (SPRY2) | O43597 |
| Fc receptor-like protein 3 (FCRL3) | Q96P31 | Protein-arginine deiminase type-2 (PADI2) | Q9Y2J8 |
| Fc receptor-like protein 6 (FCRL6) | Q6DN72 | SH2 domain-containing protein 1A (SH2D1A) | O60880 |
| Fibroblast growth factor 2 (FGF2) | P09038 | SH2B adapter protein 3 (SH2B3) | Q9UQQ2 |
| FXYD domain-containing ion transport regulator 5 (FXYD5) | Q96DB9 | Signaling threshold-regulating transmembrane adapter 1 (SIT1) | Q9Y3P8 |
| Hematopoietic lineage cell-specific protein (HCLS1) | P14317 | SRSF protein kinase 2 (SRPK2) | P78362 |
| Histamine N-methyltransferase (HNMT) | P50135 | Stanniocalcin-1 (STC1) | P52823 |
| Importin subunit alpha-5 (KPNA1) | P52294 | Stromal cell-derived factor 1 (CXCL12) | P48061 |
| Inactive dipeptidyl peptidase 10 (DPP10) | Q8N608 | T-cell-specific surface glycoprotein CD28 (CD28) | P10747 |
| Integral membrane protein 2A (ITM2A) | O43736 | Thioredoxin-dependent peroxide reductase, mitochondrial (PRDX3) | P30048 |
| Integrin alpha-6 (ITGA6) | P23229 | TNF receptor-associated factor 2 (TRAF2) | Q12933 |
| Integrin alpha-11 (ITGA11) | Q9UKX5 | TRAF family member-associated NF-kappa-B activator (TANK) | Q92844 |
| Integrin beta-6 (ITGB6) | P18564 | Transcription factor AP-1 (JUN) | P05412 |
| Interferon lambda receptor 1 (IFNLR1) | Q8IU57 | Transcription regulator protein BACH1 (BACH1) | O14867 |
| Interferon regulatory factor 9 (IRF9) | Q00978 | Triggering receptor expressed on myeloid cells 1 (TREM1) | Q9NP99 |
| Interleukin-1 receptor-associated kinase 1 (IRAK1) | P51617 | Tripartite motif-containing protein 5 (TRIM5) | Q9C035 |
| Interleukin-1 receptor-associated kinase 4 (IRAK4) | Q9NWZ3 | Tryptase alpha/beta-1 (TPSAB1) | Q15661 |
| Interleukin-5 (IL5) | P05113 | Tumor necrosis factor receptor superfamily member EDAR (EDAR) | Q9UNE0 |
| Interleukin-6 (IL6) | P05231 | Zinc finger and BTB domain-containing protein 16 (ZBTB16) | Q05516 |
| **Inflammation Panel** | | | |
| *Target* | *UniProt number* | *Target* | *UniProt number* |
| Adenosine Deaminase (ADA) | P00813 | Interleukin-15 receptor subunit alpha (IL-15RA) | Q13261 |
| Artemin (ARTN) | Q5T4W7 | Interleukin-17A (IL-17A) | Q16552 |
| Axin-1 (AXIN1) | O15169 | Interleukin-17C (IL-17C) | Q9P0M4 |
| Beta-nerve growth factor (Beta-NGF) | P01138 | Interleukin-18 (IL-18) | Q14116 |
| Caspase-8 (CASP-8) | Q14790 | Interleukin-18 receptor 1 (IL-8R1) | Q13478 |
| C-C motif chemokine 3 (CCL3) | P10147 | Interleukin-20 (IL-20) | Q9NYY1 |
| C-C motif chemokine 4 (CCL4) | P13236 | Interleukin-20 receptor subunit alpha (IL-20RA) | Q9UHF4 |
| C-C motif chemokine 19 (CCL19) | Q99731 | Interleukin-22 receptor subunit alpha-1 (IL-22 RA1) | Q8N6P7 |
| C-C motif chemokine 20 (CCL20) | P78556 | Interleukin-24 (IL-24) | Q13007 |
| C-C motif chemokine 23 (CCL23) | P55773 | Interleukin-33 (IL-33) | O95760 |
| C-C motif chemokine 25 (CCL25) | O15444 | Latency-associated peptide transforming growth factor beta-1 (LAP TGF-beta-1) | P01137 |
| C-C motif chemokine 28 (CCL28) | Q9NRJ3 | Leukemia inhibitory factor (LIF) | P15018 |
| CD40L receptor (CD40) | P25942 | Leukemia inhibitory factor receptor (LIF-R) | P42702 |
| CUB domain-containing protein 1 (CDCP1) | Q9H5V8 | Macrophage colony-stimulating factor 1 (CSF-1) | P09603 |
| C-X-C motif chemokine 1 (CXCL1) | P09341 | Matrix metalloproteinase-1 (MMP-1) | P03956 |
| C-X-C motif chemokine 5 (CXCL5) | P42830 | Matrix metalloproteinase-10 (MMP-10) | P09238 |
| C-X-C motif chemokine 6 (CXCL6) | P80162 | Monocyte chemotactic protein 1 (MCP-1) | P13500 |
| C-X-C motif chemokine 9 (CXCL9) | Q07325 | Monocyte chemotactic protein 2 (MCP-2) | P80075 |
| C-X-C motif chemokine 10 (CXCL10) | P02778 | Monocyte chemotactic protein 3 (MCP-3) | P80098 |
| C-X-C motif chemokine 11 (CXCL11) | O14625 | Monocyte chemotactic protein 4 (MCP-4) | Q99616 |
| Cystatin D (CST5) | P28325 | Natural killer cell receptor 2B4 (CD244) | Q9BZW8 |
| Delta and Notch-like epidermal growth factor-related receptor (DNER) | Q8NFT8 | Neurotrophin-3 (NT-3) | P20783 |
| Eotaxin (CCL11) | P51671 | Neurturin (NRTN) | Q99748 |
| Eukaryotic translation initiation factor 4E-binding protein 1 (4E-BP1) | Q13541 | Oncostatin-M (OSM) | P13725 |
| Fibroblast growth factor 21 (FGF-21) | Q9NSA1 | Osteoprotegerin (OPG) | O00300 |
| Fibroblast growth factor 23 (FGF-23) | Q9GZV9 | Programmed cell death 1 ligand 1 (PD-L1) | Q9NZQ7 |
| Fibroblast growth factor 5 (FGF-5) | P12034 | Protein S100-A12 (EN-RAGE) | P80511 |
| Fibroblast growth factor 19 (FGF-19) | O95750 | Signaling lymphocytic activation molecule (SLAMF1) | Q13291 |
| Fms-related tyrosine kinase 3 ligand (Flt3L) | P49771 | SIR2-like protein 2 (SIRT2) | Q8IXJ6 |
| Fractalkine (CX3CL1) | P78423 | STAM-binding protein (STAMBP) | O95630 |
| Glial cell line-derived neurotrophic factor (GDNF) | P39905 | Stem cell factor (SCF) | P21583 |
| Hepatocyte growth factor (HGF) | P14210 | Sulfotransferase 1A1 (ST1A1) | P50225 |
| Interferon gamma (IFN-gamma) | P01579 | T cell surface glycoprotein CD6 isoform (CD6) | P30203 |
| Interleukin-1 alpha (IL-1 alpha) | P01583 | T-cell surface glycoprotein CD5 (CD5) | P06127 |
| Interleukin-2 (IL-2) | P60568 | T-cell surface glycoprotein CD8 alpha chain (CD8A) | P01732 |
| Interleukin-2 receptor subunit beta (IL-2RB) | P14784 | Thymic stromal lymphopoietin (TSLP) | O969D9 |
| Interleukin-4 (IL-4) | P05112 | TNF-beta (TNFB) | P01374 |
| Interleukin-5 (IL5) | P05113 | TNF-related activation-induced cytokine (TRANCE) | O14788 |
| Interleukin-6 (IL6) | P05231 | TNF-related apoptosis-inducing ligand (TRAIL) | P50591 |
| Interleukin-7 (IL-7) | P13232 | Transforming growth factor alpha (TGF-alpha) | P01135 |
| Interleukin-8 (IL-8) | P10145 | Tumor necrosis factor (Ligand) superfamily, member 12 (TWEAK) | O43508 |
| Interleukin-10 (IL10) | P22301 | Tumor necrosis factor (TNF) | P01375 |
| Interleukin-10 receptor subunit alpha (IL-10RA) | Q13651 | Tumor necrosis factor ligand superfamily member 14 (TNFSF14) | O43557 |
| Interleukin-10 receptor subunit beta (IL-10RB) | Q08334 | Tumor necrosis factor receptor superfamily member 9 (TNFRSF9) | Q07011 |
| Interleukin-12 subunit beta (IL-12B) | P29460 | Urokinase-type plasminogen activator (uPA) | P00749 |
| Interleukin-13 (IL-13) | P35225 | Vascular endothelial growth factor A (VEGF-A) | P15692 |
| **Metabolism Panel** | | | |
| *Target* | *UniProt number* | *Target* | *UniProt number* |
| Adenosylhomocysteinase (AHCY) | P23526 | Large proline-rich protein BAG6 (BAG6) | P46379 |
| Adhesion G protein-coupled receptor E2 (ADGRE2) | Q9UHX3 | Leucine-rich repeats and immunoglobulin-like domains protein 1 (LRIG1) | Q96JA1 |
| Adhesion G-protein coupled receptor G2 (ADGRG2) | Q8IZP9 | Leukocyte immunoglobulin-like receptor subfamily A member 5 (LILRA5) | A6NI73 |
| Amyloid-like protein 1 (APLP1) | P51693 | Low-density lipoprotein receptor-related protein 11 (LRP11) | Q86VZ4 |
| Angiopoietin-2 (ANGPT2) | O15123 | Lysophosphatidic acid phosphatase type 6 (ACP6) | Q9NPH0 |
| Angiopoietin-related protein 1 (ANGPTL1) | O95841 | Meprin A subunit beta (MEP1B) | Q16820 |
| Angiopoietin-related protein 7 (ANGPTL7) | O43827 | Meteorin-like protein (METRNL) | Q641Q3 |
| Annexin A4 (ANXA4) | P09525 | Multiple coagulation factor deficiency protein 2 (MCFD2) | Q8NI22 |
| Annexin A11 (ANXA11) | P50995 | NAD kinase (NADK) | O95544 |
| Appetite-regulating hormone (GHRL) | Q9UBU3 | Nectin-2 (NECTIN2) | Q92692 |
| Arginase-1 (ARG1) | P05089 | Neural proliferation differentiation and control protein 1 (NPDC1) | Q9NQX5 |
| Aromatic-L-amino-acid decarboxylase (DDC) | P20711 | Neuronal pentraxin receptor (NPTXR) | O95502 |
| B-cell antigen receptor complex-associated protein beta chain (CD79B) | P40259 | Nodal modulator 1 (NOMO1) | Q15155 |
| Cadherin-2 (CDH2) | P19022 | N-terminal prohormone brain natriuretic peptide (NT-proBNP) | NA |
| Cadherin-related family member 5 (CDHR5) | Q9HBB8 | Paired immunoglobulin-like type 2 receptor beta (PILRB) | Q9UKJ0 |
| Calsyntenin-2 (CLSTN2) | Q9H4D0 | Peptidyl-prolyl cis-trans isomerase FKBP4 (FKBP4) | Q02790 |
| Carbonic anhydrase 13 (CA13) | Q8N1Q1 | Phosphoprotein associated with glycosphingolipid-enriched microdomains 1 (PAG1) | Q9NWQ8 |
| Catechol O-methyltransferase (COMT) | P21964 | Pro-cathepsin H (CTSH) | P09668 |
| Cathepsin O (CTSO) | P43234 | Protein FAM3C (FAM3C) | Q92520 |
| CD2-associated protein (CD2AP) | Q9Y5K6 | Protein phosphatase inhibitor 2 (PPP1R2) | P41236 |
| Chordin-like protein 2 (CHRDL2) | Q6WN34 | Protein S100-P (S100P) | P25815 |
| Clusterin-like protein 1 (CLUL1) | Q15846 | Regenerating islet-derived protein 4 (REG4) | Q9BYZ8 |
| Coiled-coil domain-containing protein 80 (CCDC80) | Q76M96 | Reticulon-4 receptor (RTN4R) | Q9BZR6 |
| Crk-like protein (CRKL) | P46109 | Retinal dehydrogenase 1 (ALDH1A1) | P00352 |
| C-type lectin domain family 5 member A (CLEC5A) | Q9NY25 | Ribosyldihydronicotinamide dehydrogenase [quinone] (NQO2) | P16083 |
| CXADR-like membrane protein (CLMP) | Q9H6B4 | Scavenger receptor cysteine-rich domain-containing group B protein (SSC4D) | Q8WTU2 |
| Diablo homolog, mitochondrial (DIABLO) | Q9NR28 | Sclerostin (SOST) | Q9BQB4 |
| Dihydropteridine reductase (QDPR) | P09417 | Semaphorin-3F (SEMA3F) | Q13275 |
| Dipeptidyl peptidase 2 (DPP7) | Q9UHL4 | Serpin B6 (SERPINB6) | P35237 |
| Disabled homolog 2 (DAB2) | P98082 | Serpin B8 (SERPINB8) | P50452 |
| DNA-(apurinic or apyrimidinic site) lyase (APEX1) | P27695 | Sialic acid-binding Ig-like lectin 7 (SIGLEC7) | Q9Y286 |
| Ectonucleoside triphosphate diphosphohydrolase 5 (ENTPD5) | O75356 | Sialomucin core protein 24 (CD164) | Q04900 |
| Ectonucleotide pyrophosphatase/phosphodiesterase family member 7 (ENPP7) | Q6UWV6 | Soluble calcium-activated nucleotidase 1 (CANT1) | Q8WVQ1 |
| Eosinophil cationic protein (RNASE3) | P12724 | Sulfatase-modifying factor 2 (SUMF2) | Q8NBJ7 |
| Fc receptor-like protein 1 (FCRL1) | Q96LA6 | Synaptosomal-associated protein 23 (SNAP23) | O00161 |
| Fructose-1,6-bisphosphatase 1 (FBP1) | P09467 | Syndecan-4 (SDC4) | P31431 |
| Galanin peptides (GAL) | P22466 | T-cell surface glycoprotein CD1c (CD1C) | P29017 |
| Gamma-enolase (ENO2) | P09104 | Thimet oligopeptidase (THOP1) | P52888 |
| Glutaredoxin-1 (GLRX) | P35754 | Thioredoxin domain-containing protein 5 (TXNDC5) | Q8NBS9 |
| GRB2-related adapter protein 2 (GRAP2) | O75791 | Thymidine phosphorylase (TYMP) | P19971 |
| Hepatoma-derived growth factor (HDGF) | P51858 | Thyrotropin subunit beta (TSHB) | P01222 |
| Inactive tyrosine-protein kinase transmembrane receptor ROR1 (ROR1) | Q01973 | Trefoil factor 2 (TFF2) | Q03403 |
| Insulin-like growth factor-binding protein-like 1 (IGFBPL1) | Q8WX77 | Tubulointerstitial nephritis antigen-like (TINAGL1) | Q9GZM7 |
| Integrin beta-7 (ITGB7) | P26010 | Tyrosine-protein kinase receptor TYRO3 (TYRO3) | Q06418 |
| Kallikrein-10 (KLK10) | O43240 | Ubiquitin carboxyl-terminal hydrolase 8 (USP8) | P40818 |
| Kynurenine-oxoglutarate transaminase 1 (KYAT1) | Q16773 | Versican core protein (VCAN) | P13611 |
| **Organ Damage Panel** | | | |
| *Target* | *UniProt number* | *Target* | *UniProt number* |
| 5'-AMP-activated protein kinase subunit beta-1 (PRKAB1) | Q9Y478 | Mevalonate kinase (MVK) | Q03426 |
| Adhesion G-protein coupled receptor G1 (ADGRG1) | Q9Y653 | Mitogen-activated protein kinase kinase kinase kinase 5 (MAP4K5) | Q9Y4K4 |
| Aldehyde dehydrogenase, dimeric NADP-preferring (ALDH3A1) | P30838 | Mothers against decapentaplegic homolog 1 (SMAD1) | Q15797 |
| Anterior gradient protein 2 homolog (AGR2) | O95994 | NAD-dependent protein deacylase sirtuin-5, mitochondrial (SIRT5) | Q9NXA8 |
| Apoptosis-inducing factor 1, mitochondrial (AIFM1) | O95831 | NEDD8 ultimate buster 1 (NUB1) | Q9Y5A7 |
| B-cell scaffold protein with ankyrin repeats (BANK1) | Q8NDB2 | Neutrophil cytosol factor 2 (NCF2) | P19878 |
| BH3-interacting domain death agonist (BID) | P55957 | Nibrin (NBN) | O60934 |
| BMP and activin membrane-bound inhibitor homolog (BAMBI) | Q13145 | Nitric oxide synthase, endothelial (NOS3) | P29474 |
| Calcitonin (CALCA) | P01258 | Nucleobindin-2 (NUCB2) | P80303 |
| Calreticulin (CALR) | P27797 | Parvalbumin alpha (PVALB) | P20472 |
| Carbonic anhydrase 12 (CA12) | O43570 | Paxillin (PXN) | P49023 |
| Carbonic anhydrase 14 (CA14) | Q9ULX7 | Peptidyl-prolyl cis-trans isomerase FKBP1B (FKBP1B) | P68106 |
| Casein kinase I isoform delta (CSNK1D) | P48730 | Perilipin-1 (PLIN1) | O60240 |
| Claspin (CLSPN) | Q9HAW4 | Phosphatidylinositol 3,4,5-trisphosphate 5-phosphatase 2 (INPPL1) | O15357 |
| CMP-N-acetylneuraminate-beta-galactosamide-alpha-2,3-sialyltransferase 1 (ST3GAL1) | Q11201 | Placenta growth factor (PGF) | P49763 |
| Cocaine esterase (CES2) | O00748 | Platelet-derived growth factor C (PDGFC) | Q9NRA1 |
| Contactin-2 (CNTN2) | Q02246 | Pleiotrophin (PTN) | P21246 |
| Corticoliberin (CRH) | P06850 | Plexin domain-containing protein 1 (PLXDC1) | Q8IUK5 |
| C-type lectin domain family 1 member A (CLEC1A) | Q8NC01 | Polypeptide N-acetylgalactosaminyltransferase 10 (GALNT10) | Q86SR1 |
| C-type natriuretic peptide (NPPC) | P23582 | Probetacellulin (BTC) | P35070 |
| Desmoglein-4 (DSG4) | Q86SJ6 | Programmed cell death protein 1 (PDCD1) | Q15116 |
| Dipeptidyl aminopeptidase-like protein 6 (DPP6) | P42658 | Prolow-density lipoprotein receptor-related protein 1 (LRP1) | Q07954 |
| DNA topoisomerase 2-beta (TOP2B) | Q02880 | Proteasome subunit alpha type-1 (PSMA1) | P25786 |
| Ectonucleoside triphosphate diphosphohydrolase 2 (ENTPD2) | Q9Y5L3 | Protein amnionless (AMN) | Q9BXJ7 |
| Ectonucleoside triphosphate diphosphohydrolase 6 (ENTPD6) | O75354 | Protein enabled homolog (ENAH) | Q8N8S7 |
| EGF-like repeat and discoidin I-like domain-containing protein 3 (EDIL3) | O43854 | Protein fosB (FOSB) | P53539 |
| Enteropeptidase (TMPRSS15) | P98073 | Protein max (MAX) | P61244 |
| Epidermal growth factor-like protein 7 (EGFL7) | Q9UHF1 | Protein phosphatase 1B (PPM1B) | O75688 |
| Erbin (ERBIN) | Q96RT1 | [Pyruvate dehydrogenase [acetyl-transferring]]-phosphatase 1, mitochondrial (PDP1) | Q9P0J1 |
| Erythropoietin (EPO) | P01588 | Ras association domain-containing protein 2 (RASSF2) | P50749 |
| Fatty acid-binding protein 9 (FABP9) | Q0Z7S8 | Ras GTPase-activating protein 1 (RASA1) | P20936 |
| Forkhead box protein O1 (FOXO1) | Q12778 | Receptor-type tyrosine-protein phosphatase eta (PTPRJ) | Q12913 |
| Fructose-2,6-bisphosphatase TIGAR (TIGAR) | Q9NQ88 | Renin receptor (ATP6AP2) | O75787 |
| Hematopoietic prostaglandin D synthase (HPGDS) | O60760 | REST corepressor 1 (RCOR1) | Q9UKL0 |
| Inactive tyrosine-protein kinase 7 (PTK7) | Q13308 | Retinoic acid receptor responder protein 1 (RARRES1) | P49788 |
| Integrin beta-1-binding protein 1 (ITGB1BP1) | O14713 | Ribonucleoside-diphosphate reductase subunit M2 B (RRM2B) | Q7LG56 |
| Interferon-inducible double-stranded RNA-dependent protein kinase activator A (PRKRA) | O75569 | Serpin A9 (SERPINA9) | Q86WD7 |
| Kidney Injury Molecule (KIM1) | Q96D42 | Serum paraoxonase/arylesterase 2 (PON2) | Q15165 |
| Killer cell immunoglobulin-like receptor 3DL1 (KIR3DL1) | P43629 | Syntaxin-8 (STX8) | Q9UNK0 |
| Leukotriene A-4 hydrolase (LTA4H) | P09960 | Syntaxin-binding protein 3 (STXBP3) | O00186 |
| Linker for activation of T-cells family member 2 (LAT2) | Q9GZY6 | Troponin I, cardiac muscle (TNNI3) | P19429 |
| Lutropin subunit beta (LHB) | P01229 | Tyrosine-protein kinase Fes/Fps (FES) | P07332 |
| Macrophage erythroblast attacher (MAEA) | Q7L5Y9 | Tyrosine-protein kinase Fgr (FGR) | P09769 |
| Macrophage-capping protein (CAPG) | P40121 | Tyrosine-protein kinase Yes (YES1) | P07947 |
| Melanoma-associated antigen D1 (MAGED1) | Q9Y5V3 | Vasohibin-1 (VASH1) | Q7L8A9 |
| Methionine aminopeptidase 1 (METAP1) | P53582 | Wiskott-Aldrich syndrome protein (WAS) | P42768 |

**Supplemental Table S4.** Candidate FDA-approved drugs targeting proteins associated with the pathology of COVID-19.

| **Target** | **Drug** | **Drug indications** | **Interaction Score** | **Sources** |
| --- | --- | --- | --- | --- |
| **TGFA** | PANITUMUMAB | Antineoplastic and  immunomodulating agent | 1.47 | PharmGKB |
|  | CETUXIMAB | Antineoplastic and  immunomodulating agent | 0.72 | PharmGKB |
| **TYMP** | PROGESTERONE | Contraceptive | 0.31 | NCI |
|  | TIPIRACIL HYDROCHLORIDE | Antineoplastic agent | 10.3 | ChemblInteractions |
|  | SULFASALAZINE | Antiinflammatory agent | 0.9 | NCI |
|  | FLUOROURACIL | Antineoplastic and  immunomodulating agent | 0.39 | NCI\|PharmGKB |
|  | CAPECITABINE | Antineoplastic agent | 0.57 | PharmGKB |

**Supplemental Table S5.** Candidate FDA-approved drugs targeting proteins associated with all-cause in-ICU mortality.

| **Target** | **Drug** | **Drug indications** | **Interaction Score** | **Sources** |
| --- | --- | --- | --- | --- |
| **NQO2** | CYCLOPHOSPHAMIDE | Antineoplastic and immunomodulating agent | 0.16 | PharmGKB |
|  | MENADIONE | Antifibrinolytic agents | 0.88 | TEND |
|  | MELATONIN | Hypnotics and Sedatives | 0.32 | TTD |
|  | IMATINIB | Antineoplastic agent | 0.13 | DTC |
|  | DOXORUBICIN | Antineoplastic and immunomodulating agent | 0.13 | PharmGKB |
| **IL1A** | HYDROXYCHLOROQUINE | Antimalarial and Immunosuppresor | 1.87 | NCI |
|  | OLANZAPINE | Antipsychotic agent | 0.31 | PharmGKB |
|  | RILONACEPT | Antiinflammatory agent | 20.61 | TEND |

| **Supplemental Table S6.** Top correlations between proteins and secondary outcomes. | | |
| --- | --- | --- |
| **Protein** | **rho** | **p value** |
| ***Duration of hospital stay*** | | |
| **TRIM21** | -0.298 | 0.052 |
| **PILRB** | 0.250 | 0.105 |
| **PTPRJ** | 0.245 | 0.113 |
| **ANXA4** | 0.245 | 0.113 |
| **MCP-4** | -0.244 | 0.116 |
| ***Duration of ICU stay*** | | |
| **TRIM21** | -0.256 | 0.098 |
| **PILRB** | 0.255 | 0.098 |
| **SERPINB6** | 0.253 | 0.102 |
| **CALR** | 0.252 | 0.103 |
| **SERPINB8** | 0.240 | 0.121 |
| ***IMV duration*** | | |
| **CALR** | 0.253 | 0.111 |
| **PILRB** | 0.231 | 0.146 |
| **CST5** | -0.230 | 0.148 |
| **CD1C** | -0.228 | 0.151 |
| **MCP-2** | 0.214 | 0.179 |

# Supplementary Figures

Figure S1

**
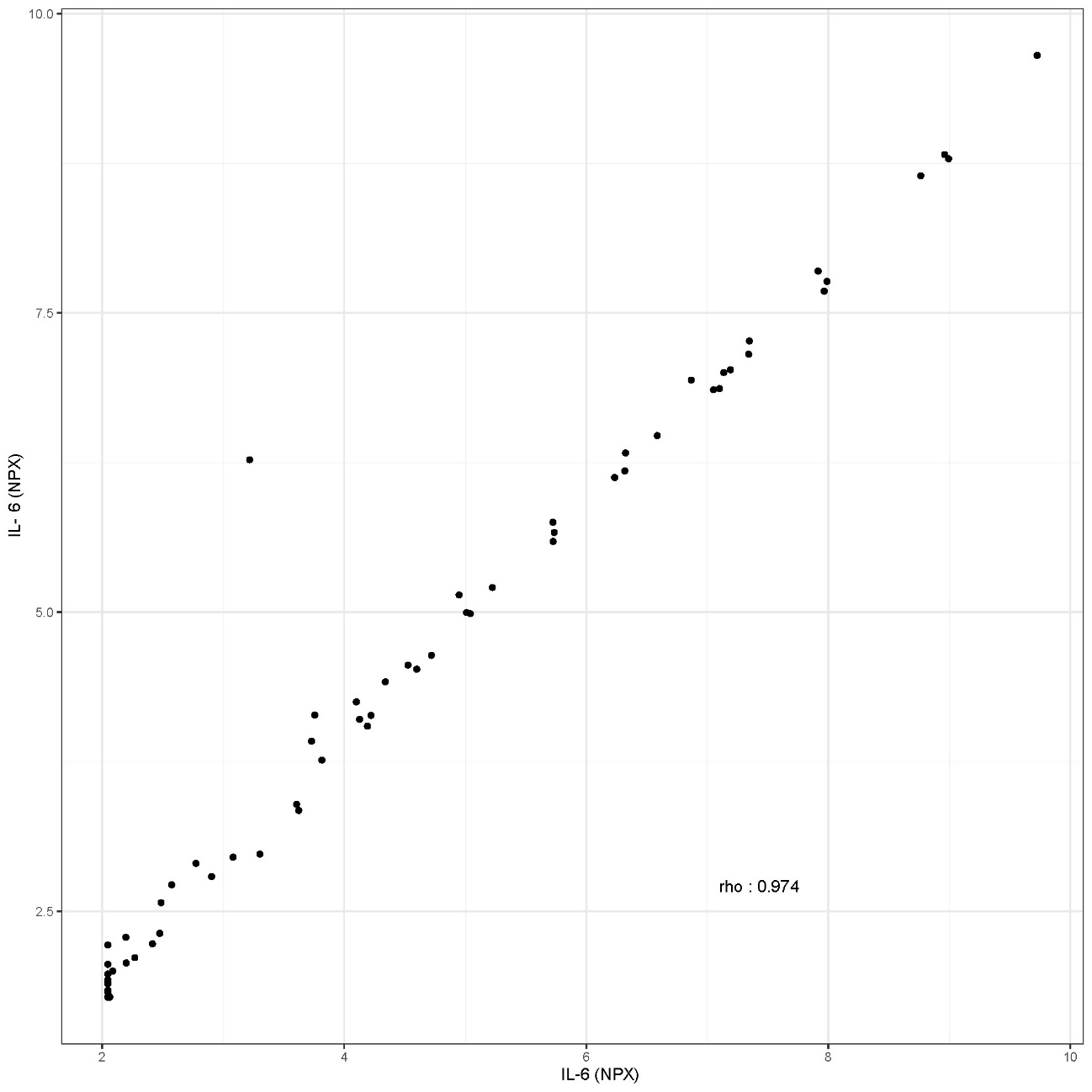
**

**Figure S1**. Correlation between IL-6 levels of the Immune Response and Inflammation Olink panels. The coefficient rho is displayed.

Figure S2.


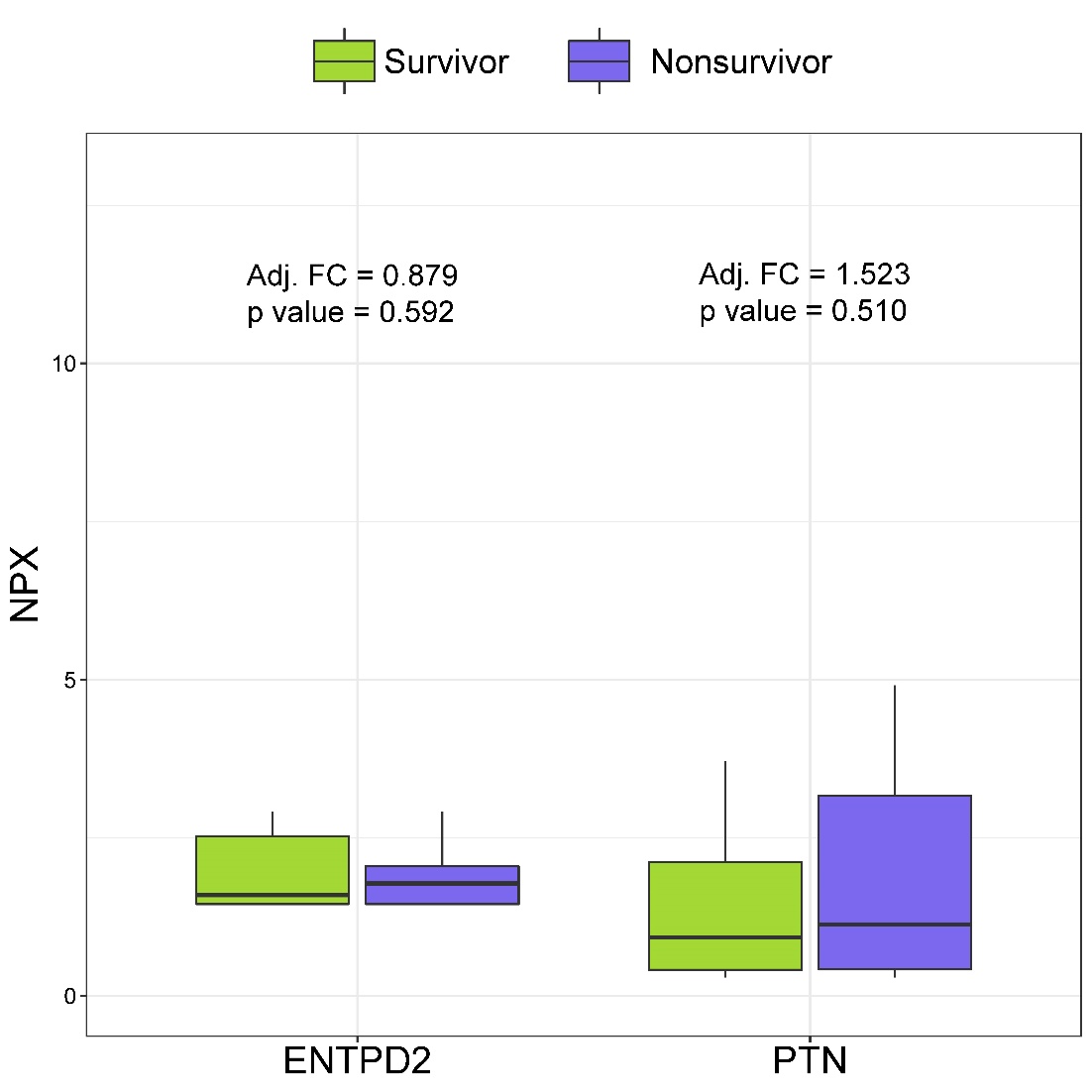


**Figure S2. Boxplot displaying PTN and ENTPD2 levels in survivors and nonsurvivors from the non-COVID-19 group.** The adjusted fold change is displayed and the significance level for each comparison is described by the p value.
